# Esportes, divertimentos e saúde em Curitiba na transição do século XIX para o XX

**DOI:** 10.1590/S0104-59702025000100007

**Published:** 2025-03-31

**Authors:** Leonardo do Couto Gomes, Wanderley Marchi

**Affiliations:** i Professor, Universidade Federal do Mato Grosso do Sul. Campo Grande – MS – Brasil; ii Professor, Programa de Pós-graduação em Educação Física/Universidade Federal do Paraná. Curitiba – PR – Brasil leonardodocoutogomes@gmail.com; iii Professor, Programa de Pós-graduação em Educação Física/Universidade Federal do Paraná. Curitiba – PR – Brasil marchijr@ufpr.br

**Keywords:** História da saúde, História do esporte, Curitiba, History of heath, History of sport, Curitiba

## Abstract

O texto discute as relações entre o estímulo das experiências esportivas e recreativas físicas e as preocupações com a saúde promovidas na cidade de Curitiba entre o final do século XIX e o início do século XX. Esse período é marcado pela emergência de artefatos e estruturas de divertimento na capital paranaense, que passaram a ser associados a noções de saúde pública. Como fontes, foram utilizados jornais de Curitiba do período destacado. Foi possível concluir que os esportes e divertimentos físicos demonstraram sintonia com ideais de modernização urbana e fortalecimento da saúde em prol da robustez da nação.

## Abstract (en)

The present text discusses the relationships between stimulating sports and physical recreational experiences and health concerns promoted in the city of Curitiba between the final years of the nineteenth century and the beginning of the twentieth century. The period is marked by the emergence of hobby artifacts and structures in the capital of Paraná state, which became associated with ideas of public health. Curitiba newspapers from the period were used as sources. It was possible to observe that sports and physical entertainment demonstrated harmony with the ideals of urban modernization and strengthening of health in favor of a robust nation.

A transição do século XIX para o XX no Brasil foi um momento de intensas intervenções de ordem modernizadora nas cidades brasileiras – essas intervenções, de modo geral, quase sempre produzidas por grupos locais, que se baseavam em símbolos europeus (Freyre, 1974; Moraes, 2001). É necessário pontuar que as transformações do período em questão também estavam em sintonia com o desejo de que a recém-pátria se transformasse em um país com “símbolos próprios”, cujos passado e tradições do antigo colonizador deveriam começar a ser superados – ainda que, por vezes, o termômetro desses anseios fosse representado pelos parâmetros provenientes do velho continente (Sevcenko, 1983).

Nesse sentido, no período em questão, a emergência de aspectos ligados a urbanização, luz elétrica, ferrovias, indústrias, telégrafos, novos aparatos tecnológicos em geral e espaços de lazer, segundo Dias et al. (2020), serviu como índice privilegiado de progresso. Dessa forma, os momentos de diversão, sobretudo os esportivos, passaram a ser considerados indicadores de modernização^1^ das cidades. Esses indicadores, portanto, deveriam ser atingidos e buscados para angariar tal *status*.

Sevcenko (1992) aponta que a interpretação sobre os espaços de lazer como elemento importante para a estruturação e modernização das cidades era visualizada em todo o país. Assim, o autor indica que, principalmente, os divertimentos^2^ desportivos representavam para a elite governante uma relevante possibilidade de auxiliar no modelamento de infraestrutura e social do Brasil. Essas práticas se constituíram em um útil meio para esse fim, visto que já estavam difundidas em cidades europeias, centros de referência de desenvolvimento urbano. Além disso, eram compreendidas também como artifícios para a promoção e o refinamento de certos costumes e atitudes, principalmente os ligados à etiqueta, à postura, ao exercício físico e à saúde da população – noções das quais o esporte se aproximava na Europa, sendo visto como um importante contribuinte para a constituição de uma sociedade mais forte e saudável (Vigarello, 2008).

Os esportes e seus espaços de difusão, nesse sentido, seriam meios profícuos para a sociedade brasileira emular gestos sentidos como contribuintes para o desenvolvimento da jovem pátria. Nessa direção, Melo e Peres (2016) e Melo (2021) observam que as dinâmicas esportivas se forjaram desde os seus primórdios em solo nacional em torno da ideia de útil e agradável *–* não eram apenas diversões, mas um contributo para a prosperidade da nação. Para o autor, essa percepção seria ainda mais enfatizada com a vinculação dos esportes aos discursos que destacavam seu potencial para a saúde – algo que se ligava a uma nova compleição muscular e a uma nova exposição do corpo, isso tudo amplamente articulado às dinâmicas físicas emergentes na época.

No caso do Rio de Janeiro, então capital federal no período estudado, a relação entre esporte, exercício físico e cuidados com o corpo evidenciava-se especialmente pela prática do remo. A dinâmica, segundo Melo (2021), seria pioneira no país em relação à propagação de visões que colocavam o praticante como portador de um conjunto de domínios ligados a ideias de determinação, honestidade, caráter, disciplina, corpo sadio e vigoroso – elementos que, gradualmente, eram considerados pela sociedade brasileira como fundamentais para a educação da mocidade.

Em São Paulo, Medeiros, Silva e Quitzau (2023) e Oliveira e Góis Júnior (2023) também observam que os mais variados esportes e divertimentos com características físicas passaram a dinamizar discursos associados a ideias de transformação do espaço urbano e de poderosos divertimentos, que desenvolviam agilidade, destreza e robusteciam os músculos, dando-lhes força e vigor. Aspectos similares são observados por Karls (2017), que explora as facetas do esporte em Porto Alegre. O autor detecta que as práticas físicas no quartel final do século XIX na capital sul-rio-grandense se colocavam cada vez mais como instrumentos aliados aos ideais de modernização e saúde.

Outras regiões, além do eixo Sul-Sudeste, também tiveram experiências semelhantes de diversão ligadas a esses discursos durante o mesmo período. Em Salvador, Machado e Rocha Júnior (2020) apontam que os caminhos pensados para o progresso na capital baiana buscavam vinculações estreitas com noções de higiene, embelezamento urbano e difusão de espaços e momentos para a prática de exercícios físicos. Os estudos de Dias (2018) e Dias e Souza (2020) observam conexões na mesma direção em Manaus, Goiás e Mato Grosso. Os autores, ao notarem as características de uma variedade de formas de entretenimento, revelam que os divertimentos influenciavam discussões sobre a organização e transformação do espaço urbano, além de benefícios físicos para aqueles que os praticavam.

Considerando que as experiências de cada local em que os esportes e divertimentos físicos se fizeram presente foram distintas, questiona-se: como essas dinâmicas teriam se organizado em Curitiba? Cidade que, assim como as citadas anteriormente, passou por intensas transformações vinculadas à circulação de noções modernistas e sanitárias nos séculos XIX e XX.

Os esportes, divertimentos físicos e seus espaços de oferta na capital paranaense são elementos visualizados nos trabalhos de Moraes e Silva (2011), e Moraes e Silva, Quitzau e Soares (2018) como contribuintes para a formação da cidade. Os autores também apontam que tais ambientes e gestos contribuíram para a difusão do apreço pelo exercício físico em Curitiba. Além disso, os pesquisadores destacam que, nessa altura, o hábito de exercitar-se começava a ser um aliado do discurso de caráter médico e se tornava, aos olhos do poder público, um importante combatente das mazelas do corpo.

Apesar de os estudos anteriores fornecerem detalhes sobre a organização dos esportes e divertimentos em Curitiba, além de reforçarem nuanças dessas atividades como um esforço ligado às ideias de caráter médico e urbanização na capital paranaense, os referidos trabalhos não focalizaram especificamente essas potencialidades, sobretudo como dinâmicas que nos possibilitam prospectar tais momentos como estratégias de estímulo à difusão de noções sanitárias.

Para alcançar o objetivo deste texto, utilizou-se como fontes periódicos publicados na capital paranaense durante o período analisado. Os jornais foram selecionados porque constituíram importantes elementos no processo de urbanização de Curitiba, divulgando discursos relacionados àquilo que se considerava moderno (Corrêa, 2009). Entre as narrativas produzidas e difundidas pela imprensa, encontravam-se os entretenimentos físicos e esportivos. Ademais, salientamos que a tipografia curitibana, nesse momento, como delimita Myskiw (2008), já se conformava como um lócus de divulgação pública, afirmando-se, portanto, como uma fértil possibilidade para compreendermos os acontecimentos do cotidiano da cidade.

Cabem algumas considerações acerca do perfil desses jornais. Fundado em 15 de março de 1886 e extinto em 1930, o jornal *A República* foi a primeira folha ligada aos preceitos republicanos na capital paranaense. Nele, eram divulgadas diversas aspirações vinculadas ao ideário republicano, tais como os discursos de ordens política e cívica, de embelezamento e desenvolvimento urbano e civilização dos costumes.

*O Munícipio* foi um órgão da municipalidade de Curitiba. De curta circulação (1897 a 1898), o periódico afirmava ter como missão informar a população curitibana de todos os acontecimentos e serviços pensados para o progresso da cidade (O Município, 4 dez. 1897, p.1). Entre os focos de publicação, destacam-se notícias sobre literatura, religião, noções de saúde e higiene pública.

O jornal *Diário da Tarde* foi um órgão de tendência liberal e anticlerical. Circulou pelas ruas de Curitiba de 1899 a 1940. Tinha como principal finalidade exigir do poder público posturas ligadas aos interesses liberais que visavam especialmente ao progresso material da cidade e à modernização dos comportamentos em sociedade.

Vejamos a seguir como as experiências de entretenimento físico promovidas em Curitiba dinamizaram questões em torno de noções de saúde.

## Uma Curitiba moderna, divertida e saudável

Os avanços pela busca da transformação urbana em Curitiba ocorriam simultaneamente ao aumento progressivo de habitantes. Nos anos finais do século XIX, a cidade contava com cerca de cinquenta mil pessoas e apresentava mais de 120 quadras (Bahls, 1998). Outro elemento central na dinamização da sociedade curitibana foi a produção da erva-mate, que passou a atingir cultivos em escalas técnico-industriais. Sua venda, de acordo com Pereira (1996), estava se expandindo para diversos países, principalmente Uruguai e Argentina. Isso foi otimizado graças à criação da estrada de ferro que ligava Paranaguá e Curitiba, em 1885. O trajeto se constituía como o principal meio de ligação entre a capital e o litoral, o que representava a possibilidade da intensificação do transporte ervateiro e, por consequência, mais eficiência e lucros. Aliada principalmente ao capital privado ervateiro, a Câmara Municipal ganhou oportunidade de remodelar a urbe.

Essas alterações teriam respaldo em discursos de embelezamento urbano, que, conforme sinaliza Bahls (1998), priorizavam a emergência de espaços inéditos e tinham o objetivo de dar fim às mazelas públicas (áreas ociosas, insalubres, não pavimentadas etc.). Em 1885, surtos epidêmicos de tifo, pneumonia, varíola e sarampo expuseram diversas preocupações relacionadas às condições de salubridade e higiene da população curitibana.

Os processos de abolição da escravidão (1888) e da Proclamação da República (1889), segundo Benvenutti (2004), também promoveram diversas alterações na estrutura sanitária e burocrática do Paraná. De acordo com o autor, as noções de saúde pública e de higiene pessoal e coletiva foram alteradas, houve a institucionalização do trabalho livre e uma maior autonomia/verba para a municipalidade. Essas movimentações possibilitaram a efetivação de uma série de renovações para a cidade.

Os espaços de divertimento são bons exemplos do avanço da urbanidade na capital paranaense. Nesse momento, eram visíveis diversos ambientes de diversão na cidade. Um exemplo é a construção do Passeio Público, ainda em 1886. Ao se apropriar de uma área pantanosa e insalubre, a Câmara Municipal, com suporte financeiro dos grandes proprietários de erva-mate, reestruturou uma área inutilizada da capital paranaense e a transformou em um espaço com condições de recreio para a população (Molina, 2020).

Esse espaço, de acordo com Gomes e Capraro (2022), se tornou um local onde se ofertava um pouco de tudo. Seria ali, segundo os mesmos autores, que diversos desejos da vida em cidade, como o *flâneur*,^3^ melhor se difundiriam. Tratava-se, portanto, de mais uma ação conjunta dos poderes público e privado ligada aos divertimentos que requintavam Curitiba, sobretudo por meio da promoção de novas infraestruturas e novos comportamentos marcados por um ideário que os considerava símbolos de novos tempos.

Entre os divertimentos ofertados no Passeio Público, destacavam-se as dinâmicas náuticas. Em Curitiba, essas experiências, de acordo com Gomes e Capraro (2022), nos anos finais dos oitocentos e início dos novecentos, não se demonstraram duradouras. Isso ocorreu porque a capital paranaense não tinha recursos hídricos marítimos, tampouco vastos lagos e rios. De toda forma, os autores detectam que tentativas de práticas ligadas a algo similar ao remo nesse período ocorreram no primeiro parque da cidade, além de indicarem que a urbe estava sintonizada às ideias vinculadas a esse esporte, sobretudo aquelas apegadas às noções de higiene, saúde e fortalecimento físico.

O passeio público ofereceria diversas atividades físicas, inclusive gratuitamente, para as crianças, conforme reforça um contrato do parque: “O contratante concede uma vez por semana, entrada gratuita a todas as escolas públicas da Capital, a fim de exercitar as crianças nos exercícios de musculatura” (A República, 8 dez. 1895, p.4.).

Notemos uma preocupação com a presença de crianças. Trata-se de um indício de que o reconhecimento de se exercitar por meio de divertimentos físicos como um elemento de educação e fortalecimento do corpo já ganhava traços de utilidade para o meio escolar. Segundo Moraes e Silva (2011), essa era uma percepção de que as atividades físicas promoveriam um benefício para toda a população, inclusive a infantil.

Em uma matéria em primeira página não assinada no jornal *A República*, foram evidenciados alguns significados que o exercício físico, por meio de divertimentos esportivos, começava a ganhar na capital paranaense nos anos finais do século XIX. Observemos:

Não se podem passar debaixo do silêncio, nem mesmo em um breve artigo, os efeitos fisiológicos dos exercícios físicos que, sem ter aplicação médica especial, têm uma influência higiênica importante. Aos nossos tempos, a cultura intelectual tem prevalecido cada vez mais, com excessivo detrimento do organismo, e quantas doenças vemos a tão deploráveis hábitos! Os exercícios físicos, os ‘sports’ em geral, quais: equitação, esgrima, canoagem, salto, corrida, marcha etc., são empregados como bom êxito em certas afecções musculares ou nervosas, mas esses são principalmente meios preventivos e profiláticos (A República, 29 abr. 1896, p.1.)

Moraes e Silva, Quitzau e Soares (2018), ao investigarem a emergência de uma cultura física junto à natureza em Curitiba no respectivo período, apontam que se exercitar se tornava, aos olhos do poder público, influenciados por discursos de caráter médico, um importante combatente das mazelas do corpo. Logo, para os autores, a construção de espaços para a prática de atividades físicas se forjava em contributos marcados por duas frentes modernizadoras – a que ofereceria sofisticação urbana e a formulação de estilos de vida considerados saudáveis.

Nessa direção, a notícia acima reforça as ponderações dos autores, afinal, ao considerarmos que o excerto foi publicado na primeira página do periódico, é possível cogitar que o debate sobre os benefícios do exercício para o corpo, de fato, ganhava ênfase. Infelizmente, não conseguimos detectar quem foi o autor do texto; porém, é um bom esclarecimento sobre algumas noções de saúde e higiene que passavam a ser atribuídas ao esporte e às atividades recreativas de caráter físico em Curitiba.

Adverte-se que essas movimentações de cunho sanitário, pensadas para o esporte e os exercícios físicos, estavam interligadas aos avanços que a medicina e, consequentemente, os discursos médicos estavam representando no período. Marcos, como a criação da Aspirina produzida pelo químico Felix Hoffmann, de origem alemã, são bons indicativos dos saltos farmacológicos presentes no final do século XIX e no início do XX (Hobsbawm, 1988). Nesse mesmo contexto, os estudos sobre as teorias de doenças transmissíveis ganham vazão em solo nacional. Com relação a esse pensamento, recebem destaque as medidas difundidas pelo médico sanitarista Oswaldo Cruz na capital federal, que, com base nos estudos pioneiros do francês Louis Pasteur acerca de bactérias, vírus e suas propagações, inicia campanhas sanitárias focadas no controle e combate de doenças, como febre amarela, peste e varíola, que frequentemente assolavam as cidades brasileiras (Chalhoub, 2006).

De fato, os médicos bacteriologistas e suas inéditas teorias de transmissibilidade e controle de doenças foram centrais para uma nova perspectiva em relação às preocupações sanitárias. Ao investirem em pensamentos ligados aos cuidados com corpo, higiene e limpeza urbana, seus discursos tiveram algum impacto na visualização dos divertimentos, exercícios físicos e esportes como contribuintes para tais questões e, por consequência, fortaleceram ainda mais suas utilidades no seio social (Dias, 2010).

Outros discursos médicos que fortaleceram os cuidados do corpo, associados ao hábito de se exercitar, foram os de caráter homeopático. No caso paranaense, segundo Fraiz (2014), essas noções foram influenciadas principalmente pelos trabalhos do médico Nilo Cairo.^4^ Apesar das diferenças de opinião entre homeopatas e bacteriologistas em grande parte das teorias médicas, havia uma sintonia quando se tratava dos benefícios do exercício físico para o fortalecimento e a melhoria da saúde. Os passeios junto à natureza, caminhando, de bicicleta, a cavalo ou até mesmo de automóvel seriam “prescrições” usuais dos homeopatas, que viam a realização dessas práticas em contato com o ar puro como um útil método de cura (Fraiz, 2014). É necessário considerar que esses pensamentos estavam veementemente atrelados a uma embrionária ciência médica que estava se estruturando no Brasil dos meados do século XIX, por meio da instalação das pioneiras escolas de medicina do país (Schwarcz, 1993).

No caso curitibano, se o remo não teria logrado sucesso em virtude das condições hídricas da capital paranaense, as bicicletas começariam a tomar as ruas da cidade e a enfatizar a relação entre as práticas de caráter físico e as melhorias para o corpo. As dinâmicas ciclistas, conforme aponta Góis Júnior (2013), obtiveram popularidade no Brasil ainda no fim do século XIX, estando associadas diretamente a representações de avanço tecnológico, rapidez, vertigem, mobilidade, inovação e prática esportiva. Nos jornais de Curitiba, é possível detectar alguns significados atribuídos ao artefato, sobretudo aqueles ligados aos cuidados e à manutenção da saúde:

Efeitos da bicicleta sobre os órgãos

Os médicos desconfiaram de que o ciclismo poderia influir, mais ou menos no organismo. Numerosos trabalhos se têm publicado neste sentido. Com emprego do ‘fanendoscópio’ obtiveram a completa e rápida reprodução dos órgãos internos. Examinaram sucessivamente três ciclistas, e desenharam seus órgãos antes da corrida, imediatamente depois e durante muitos dias consecutivos. A comparação das imagens levou-os às conclusões seguintes: Os órgãos diminuíram, muito especialmente os abdominais, o baço, o fígado e o estômago. Sua ação sobre o coração é certamente menos violenta e menos prejudicial que a de todos os exercícios musculares (Diário da Tarde, 16 out. 1900, p.1).

A fonte relata os possíveis efeitos das experiências ciclísticas para os órgãos, evidenciando ainda percepções médicas voltadas para a possibilidade de o esforço físico ser um contributo para um melhor funcionamento do corpo. A ligação entre esporte e benefícios para a saúde estava em pauta. Seria um novo discurso no qual os anseios progressistas se alinhariam, especialmente porque eram debatidos como proveitosos para o fortalecimento físico da população, algo que viria a contribuir diretamente para a prosperidade de uma nação mais forte. A continuidade da análise jornalística fornece ainda mais detalhes da associação entre a prática ciclística e os benefícios para a saúde: “Os pulmões acham nesta maneira de progressão um exercício salutar e uma dilatação favorável. Os músculos utilizados pelas bicicletas são muito mais numerosos que os utilizados pela marcha. Resulta disso que a bicicleta fornece um exercício eminentemente favorável ao desenvolvimento muscular” (Diário da Tarde, 16 out. 1900, p.1).

Os exercícios físicos e esportivos, como bem destacam Mendes e Nóbrega (2008), Melo e Peres (2014) e Dalben e Góis Júnior (2018), ganhavam, no período, entonação de serem autênticos protetores da saúde corporal; seriam uma espécie de “divulgadores” das preocupações higiênicas e de regeneração da raça. A nota a seguir evidencia ainda mais essa relação: “Nota-se com satisfação que de um tempo para cá o nosso povo vai tomando gosto pelos divertimentos esportivos, caracterizando-se extraordinariamente o gosto pela bicicleta que tantos serviços tem prestado à educação física de nossa mocidade. Atualmente já se vê muitos rapazes musculosos, robustos, com cores sadias e alegres” (A República, 28 nov. 1901, p.1).

A observação jornalística deixa claro que praticar divertimentos físicos começava a se tornar uma tendência entre a população. Mais evidente ainda é a ênfase dada às vantagens de se realizar tais gestos, sendo reconhecidos com uma genuína prestação de serviço para a educação física da mocidade. Tratava-se de mais um indicador da influência que o pensamento médico passava a exercer na regulação e nos incentivos para os novos usos do corpo. Em outro termo de contrato de concessão do Passeio Público de Curitiba, os divertimentos de caráter físico demonstraram ser pauta obrigatória:

A Câmara Municipal concede ao contratante Antonio Matheus Dias Fernandes, pelo prazo de três anos, a conta do dia 1º Maio próximo, o direito de estabelecer dentro do Passeio, todos os divertimentos públicos permitidos por Lei, como sejam; Tiro ao alvo, bicicletas, ginástica, jogos malabares e atléticos, boliche, críquete, *foquet*, fantoches, regatas, corrida a pé, luta romana, florete, esgrima, natação e outros divertimentos para a instrução física e distração da mocidade, não podendo absolutamente, sob pena de multa e rescisão deste contrato, estabelecer com a denominação dos divertimentos acima citados, outros contrários à moral e aos bons costumes (A República, 1 maio 1896, p.1).

Observemos a gama de divertimentos, com características esportivas, descritos como permitidos por lei. Não foi possível detectar nos códigos de posturas, instruções públicas ou legislações da cidade algum marco legal que descrevesse precisamente a base de tais prescrições. Ao que parece, basicamente julgavam-se úteis as atividades de diversão que apresentassem potencial para a instrução da juventude. Ou seja, aquelas que demonstrassem capacidade de desenvolvimento e fortalecimento físico, além da possibilidade de afastar-se de entretenimentos associados a hábitos perniciosos, como o consumo de bebidas, violência e ociosidade.

Esse é um indício, aliás, do papel pedagógico que os divertimentos passaram a representar na sociedade. Não sabemos ao certo se todos esses divertimentos chegaram a ser ofertados no Passeio Público de Curitiba. De todo modo, são indicadores do pensamento acerca do papel instrutivo que as atividades físicas de diversão despertavam e do quanto os novos divertimentos estavam sendo irradiados na capital paranaense.

Outros dois ramos de entretenimento físico que ganharam força em Curitiba nos anos finais do século XIX e no início do século XX foram as casas de jogos da pelota basca e de patinação. Sobre a primeira modalidade, dois espaços foram centrais em sua disseminação: o inaugural Frontão Turíbio e seu sucessor, o Frontão Curitibano. Ambos estavam localizados na rua Aquidaban (atualmente Emiliano Perneta), na região central da cidade. A atividade, inclusive, contribuiu para a valorização de determinadas proezas atléticas, conforme reforça uma crônica ao abordar um dia de competições:

Desde que começou a luta e, de um lado, vimos 21 pontos e de outro 25, ficamos indecisos não sabendo quais seriam os vitoriosos, apesar de que Arissala estava bastante caipora. Etulain que foi quem se bateu com todo o vigor; na verdade foi este pelotário quem nos admirou pela sua tenaz resistência, pois seu companheiro estava mal e ainda assim lutava com muito ardor para alcançar a vitória. Larza, como já dissemos no artigo passado, mostrou-se um velho incansável, e vimos também ontem comandando os seus dois companheiros e ao mesmo tempo fazendo saques rápidos, alcançou o triunfo e deixou seus adversários em 31 pontos para 40. Abando, como nunca esperávamos, jogou de uma maneira tal, pois defendeu seu posto corretamente. João também defendeu seu posto, mas errou algumas pelotas. De modo que, para assistirmos a outra luta igual, esperamos que nos dará o prazer no próximo domingo, o digno sr. gerente (A República, 16 mar. 1898, p.3).

As considerações do cronista quanto às valências físicas observadas na disputa da modalidade, como o vigor dos praticantes, sua precisão, sua resistência, sua velocidade e alguns aspectos táticos de seus movimentos, são significativas ponderações de que as proezas atléticas de fato estavam começando a ganhar destaque e apreciação.

Quanto à patinação, Gomes, Moraes e Melo (2020), ao estudarem as experiências desse divertimento em Curitiba, que ocorreram entre 1879 e 1916, evidenciam diversas facetas da prática, percebendo que a dinâmica logrou interesse a ponto de existirem diversos rinques para a atividade espalhados pela cidade. Ofertavam-se aulas e professores para ensinar a modalidade. Assim, os patins se tornaram outra forma de entretenimento que mobilizou a capital paranaense, o que serviu como uma ferramenta educativa e impulsionou representações relacionadas às práticas recreativas de caráter físico. Um noticiarista afirmava que a patinação era “um verdadeiro e edificante torneio de instrução e de educação física e moral” (O Município, 2 jul. 1898, p.1).

Agremiações ciclísticas também começaram a ser formuladas. As corridas e os passeios de bicicleta começavam a ser uma realidade nas ruas da capital paranaense; contudo, a cidade não tinha estrutura específica para essas dinâmicas – os velódromos ainda não eram espaços existentes. Por vezes, as “máquinas de duas rodas” buscaram as dependências do Jockey Club Paranaense para a sua difusão:

Merecem um bravo caloroso os distintíssimos jovens que iniciaram ontem no Prado Paranaense interessantíssimas corridas de bicicletas. Este gênero esportivo está geralmente divulgado por todas as capitais civilizadas. A concorrência não foi grande, devido talvez ao fato de não ter sido bastante anunciada a bela e humanitária diversão. Estamos certos, porém, que outras corridas atrairão muito maior número de pessoas a quem interessam as festas elegantes e úteis. ... Os vitoriosos foram muito aclamados pelo público, terminando as corridas às 4 horas da tarde, sempre debaixo do maior entusiasmo. O benefício das entradas e pules reverteram em favor do Hospital de Caridade, sendo por tal motivo credora de toda gratidão os dignos moços que tiveram a feliz lembrança de introduzir tão belo divertimento em nossos costumes. Para o futuro serão consideravelmente animadoras tais festas, pois a boa notícia da primeira experiência correrá rápida trazida por quantos tiveram a ventura de assisti-la (Diário da Tarde, 23 set. 1901, p.2).

Notemos que o jornal recebeu a implementação desse novo gênero esportivo com bons olhos, o que enfatiza a percepção de que era algo difundido em capitais civilizadas; isso reforça a relação entre o esporte e o discurso progressista. Percebamos que as dinâmicas ciclísticas seguiram certos rituais comuns à prática das corridas de cavalos, como a cobrança de entradas e até mesmo de pules (apostas), que, nesse caso, foi convertida em benefício ao Hospital de Caridade. Essa medida foi, provavelmente, realizada com o propósito de angariar interessados para um estilo de esporte ainda pouco comum que, conforme a fonte reforça, estava começando a atrair praticantes e espectadores.

De maneira frequente, as novas dinâmicas esportivas passariam a utilizar o prado de corridas de cavalos para a sua propagação. Ao que parece, consistia-se em uma apropriação de um espaço avistado como apropriado para as experiências esportivas, não apenas pelo lado infraestrutural, no qual as bicicletas aproveitavam a pista dos equinos, mas também pelo público presente, que já estava acostumado com contemplação esportiva e demonstrava interesse por ela. Esses aspectos certamente facilitavam o angariamento de público e um bom funcionamento das corridas ciclísticas, uma vez que esses espectadores detinham comportamentos específicos para essa cena. Provavelmente, foram incorporados os gestos propagados no turfe – os modos de torcer, perder, apostar, vestir, fiscalizar, controlar e se portar durante o esporte.

Os eventos ciclísticos, de fato, foram uma das práticas esportivas que mais buscaram as dependências do Jockey Club do Paraná. As páginas jornalísticas passaram a dividir as observações sobre os momentos dos equinos e dos artefatos de duas rodas, que não raro passaram a ser realizados nos mesmos dias: “Na reunião do Club de corridas, efetuada anteontem, foram inscritos cinco páreos para as corridas do dia 18, bem como cinco páreos de bicicletas. Iniciou-se atraente festa, às 11 horas e meia, com soberba corrida dos *cyclemen’s*, na distância de 3.200 metros, venceu-a o amador Gustavo” (Diário da Tarde, 6 jun. 1899, p.1).

Webber (1988) aponta que a bicicleta, máquina relacionada a ideias de liberdade e velocidade, fora comparada com os cavalos, passando a ser um meio de locomoção mais acessível, chegando às camadas mais populares francesas e propagando-se em todos os segmentos sociais. Efetivamente, os valores das bicicletas eram menores do que os de um equino (especialmente em relação àqueles com certas melhorias genéticas ou de doma). Nos jornais, é possível detectar anúncios de venda que trazem suporte para tal comparação:

Vende-se uma especial Bicicleta, marca ‘Durkopp’ completamente nova, tendo apenas quatro meses de uso, e o seu estado é perfeito. O preço é de 280$000 (A República, 17 dez. 1903, p.3).

Troca-se com um animal novo de sela com o respectivo arreamento uma bicicleta da marca afamada (Durkopp), a qual custou na fábrica 425$000, e está atualmente em bom estado. As borrachas são novas e o selim privilegiado etc. (Diário da Tarde, 30 out. 1903, p.3).

As bicicletas importadas da marca alemã Durkopp custavam preços similares ao de um cavalo comum de transporte. É bem provável, portanto, que um equino de corrida com melhorias genéticas e de doma tivesse valor de mercado superior. Assim sendo, as bicicletas poderiam ser um artefato mais acessível em comparação aos cavalos de corrida. Comparações entre as “magrelas” e os quadrúpedes seguiram sendo elaboradas:

Publicou-se em Paris a estatística mensal, correspondente ao mês de Outubro do ano passado, dos acidentes ocorridos em França, causados pelo cavalo, pela bicicleta, pelo automóvel e pelas estradas de ferro. Pesam sobre a consciência do cavalo 967 acidentes, dos quais 82 mortes e 885 ferimentos; a bicicleta causou 119 acidentes, dos quais 6 mortes e 118 ferimentos; o automóvel, 88 acidentes, sendo 2 mortes e 86 feridos, as estradas de ferro, 145 acidentes, dos quais 8 mortes e 137 ferimentos. De tudo isso resulta que foi o cavalo, entre os diversos agentes de locomoção, o que maior número de crimes cometeu contra a humanidade. E foi o cavalo a mais nobre conquista do homem. Ele vinga-se (A República, 1 out. 1901, p.2).

O relato enfatiza a estatística mensal de acidentes ocorridos na França relacionados aos quatro principais meios de locomoção do início do século XX. Observemos que a bicicleta, o automóvel e o trem ganhavam nos números como artefatos mais seguros do que o cavalo. O equino, que até então reinava como um importante símbolo de trabalho, deslocamento e esporte, começava a perder espaço para as inéditas invenções. Na corrida pelo progresso, as novas invenções avançavam em disparada, apoiadas por discursos ligados a ideias de maximização do tempo, segurança ou, então, pela melhoria da saúde – noções em que os quadrúpedes ficavam para trás e às quais nada se vinculavam.

A ênfase dada ao discurso sobre os benefícios do esporte e do exercício físico para o cuidado do corpo era tão grande que, frequentemente, alguns gêneros de diversão passavam a atribuir expressões dessa natureza. Práticas, como o jogo da pelota basca, com frequência eram anunciadas como um “útil e higiênico exercício” (A República, 16 set. 1896, p.1). Até mesmo as touradas buscariam essa associação. Um divulgador assim afirmou: “Em grande número de países da Europa civilizada, aquela arte é considerada como um meio fácil de desenvolver a musculatura do homem, adaptando-o assim a todos os misteres materiais da vida” (A República, 8 dez. 1895, p.4.).

Os divulgadores, pelo visto, pretendiam sintonizar as dinâmicas aos novos tempos, vinculando-as, assim, a uma locução em ascensão na sociedade – algo que, por consequência, as afastaria das possíveis visões de que seriam divertimentos perniciosos para a vida pública. No caso da pelota basca, em Curitiba, a atividade, segundo Gomes e Moraes (2022), sofria críticas quanto à sua moralidade. Era um jogo rotineiramente associado a brigas e bebidas, e que, conforme os autores, buscava aproximar-se dos discursos de higiene e saúde, justamente para se distanciar de certos costumes considerados imorais.

Características similares são observadas nas touradas da capital paranaense. De acordo com Melo e Gomes (2021), o entretenimento penava por ser um costume julgado como bárbaro e ligado a tradições portuguesas que precisavam ser superadas. Dessa maneira, algumas diversões passavam a ser mais valorizadas por seus possíveis benefícios sociais e suas relações com a saúde, isto é, passaram a ser mais reconhecidas por influência na saúde corporal do praticante e pela difusão de costumes afáveis, e não pelo caráter de jogo ou divertimento desinteressado.

Em meio a essa atmosfera de busca pela relação dos divertimentos físicos com os discursos de utilidade sanitária, outras agremiações e outros gêneros esportivos também estavam conformando-se em Curitiba; a associação com os benefícios médicos e higiênicos de tais práticas ficavam ainda mais evidentes. É o caso do Sport Club, fundado em 1904, uma instituição especializada em diversos esportes:

Sport Club – Um grupo de jovens desta cidade, interpretando o sentimento da sociedade moderna, qual o da prática de exercícios físicos, regulados pela higiene, resolveu fundar uma associação, à qual denominam Sport Club. Declarou que esta nova sociedade se comprometia a estabelecer em sua sede social tudo o que julgasse conveniente ao desenvolvimento físico, proporcionando aos sócios, durante as horas mais apropriadas do dia, os seguintes jogos: futebol, baseball, *laron-tenis*, críquete, *skating-rink*, pela, esgrima, ciclismo, ginástica; exercícios esses usados como vantagem nas mais adiantadas cidades do mundo, reconhecidos como os mais eficazes pela higiene moderna (Diário da Tarde, 26 set. 1904, p.2).

A notícia jornalística é clara: o clube visualizava que poderia contribuir com a difusão de hábitos de saúde e higiene por meio dos mais distintos exercícios físicos. Vale observar a ligação da instituição com um discurso cuja finalidade era, mediante o esporte, contribuir com o progresso e o processo civilizatórios da capital paranaense. Para Souza (2014), esses elementos em torno do esporte ofereciam à instituição e àqueles que se envolviam a outorga do que seria uma sociedade moderna na época. Dessa maneira, os fundadores da agremiação adquiriam a condição de intérpretes da modernização desejada, uma espécie de vanguarda, capaz de capturar o “espírito do tempo” e convertê-lo em prática.

Reparemos no leque de esportes ofertado pelo Sport Club. Destaque para as atividades inéditas, especialmente de origem britânica,^5^ como tênis, críquete e principalmente futebol. De acordo com Moletta Junior (2009), esse relato de experiência foi uma das primeiras menções ao esporte bretão no Paraná.

Entre 1890 e 1913, houve uma acentuada intervenção britânica no Brasil nos aspectos culturais, como no caso de alguns esportes, e principalmente no cenário econômico. De acordo com os levantamentos de Singer (1975), nesse período os britânicos investiriam cerca de 224 milhões de libras em solo nacional. Esses investimentos ocorriam em várias áreas, principalmente por meio de empréstimos ou estabelecimento de subsidiárias e empresas destinadas a fornecer serviços comuns na Europa. Vale destacar os investimentos no setor de transporte ferroviário, bondes e navegação.

Os novos e variados esportes podem ser o motivo que justifica o fato de um colunista afirmar que o Sport Club disponibilizava “elegantes diversões e úteis torneios de força, destreza e agilidade, bem diversos dos entretenimentos anti-higiênicos e atrofiantes generalizados nesta capital” (A República, 28 nov. 1904, p.2).

Na verdade, a valorização de espaços e atividades que poderiam promover exercício físico era celebrada principalmente porque representava a possibilidade de se divertir e, ao mesmo tempo, afastar-se de costumes descorteses, além de cuidar da saúde da população. A crônica anônima a seguir reforça esses pontos:

Essa tendência pela bicicleta que vai dia a dia se generalizando, dá-nos consoladora esperança de ver, em breve, surgir do meio dessa população anêmica uma geração bela e vigorosa. Se desgraçadamente ainda alguns moços vão perder a saúde, a inteligência e até a própria mocidade nas inúmeras casas de jogos que infestam a capital, outros – e estes felizmente em maior número – dedicam-se aos jogos atléticos, aos divertimentos esportivos que têm a grande vantagem de reunir o útil ao agradável (A República, 28 nov. 1901, p.1).

Os divertimentos esportivos e jogos atléticos exemplificavam a combinação do útil com o agradável, pois proporcionavam distração e euforia, ao mesmo tempo que contribuíam para o fortalecimento e a recuperação da saúde. No entanto, essa não era sua única função. Passavam a ser proveitosos também por ser uma oposição a eventuais atividades recreativas que se vinculavam à jogatina e ao vício – costumes tidos como imorais e que, de acordo com Moraes e Silva (2011), eram associados à possibilidade de conduzir para a desordem, a violência e a degeneração da sociedade curitibana.

Com o comparecimento de novas instituições, como as esportivas, as narrativas do espaço social e do mercado de divertimento paranaense se metamorfosearam ainda mais. À medida que avançavam as manifestações recreativas físicas e esportivas ligadas aos cuidados do corpo e fortalecimento físico, progrediram também os discursos relacionados à disciplina e à honestidade, que também passaram a ser incorporados por esses espaços e momentos.

Jogos de azar envolvendo dinheiro (aqueles em que os sujeitos dependiam exclusivamente da sorte para ganhar), como os de baralho e dados, e outras práticas que fossem associadas a baderna, jogatina e vícios, deveriam ser fiscalizados e até mesmo excluídos da sociedade. Para serem considerados úteis para a vida social, esses divertimentos deveriam personificar valores físicos e éticos. Seria somente por meio do esforço físico e da competição saudável e honesta que os praticantes e espectadores desenvolveriam virtudes como a perseverança, a lealdade e a justiça (Melo, 2021).

Boni (1985) lembra que, nesse período, parâmetros morais ligados a ideias de civilidade, bons comportamentos públicos e repúdio à selvageria e à violência se estabeleciam como princípios fundamentais na capital paranaense. Segundo a autora, eram aspectos vistos como contribuintes para uma cidade que ambicionava a melhoria da sua conformação urbana; os esportes e divertimentos, por sua vez, também deveriam seguir essa direção. A imprensa deixava claro os proveitos das dinâmicas esportivas quando comparadas aos jogos perniciosos:

Não se deve confundir o jogo com jogos; se um atrofia o coração, a saúde, o entendimento, o outro augura o corpo, desperta o raciocínio, reforça a inteligência. Infelizmente um tem sido a morte do outro. A posição adstrita aos frequentadores do pano verde instila os músculos para os ativos exercícios do esporte. Os primeiros jogos que inventaram os homens foram a luta, os cestos, a clava, a lança, a pela, o troia, o lançar a barra, o ferir o alvo com a seta, o correr a cavalo no estádio, o saltar as valas, o nadar vestido de armas e outros semelhantes; cujo exercício, diz um escritor, era tão útil para a saúde e robustez do corpo, como necessário para a guerra, para a agricultura e para os outros trabalhos da família e da pátria (Diário da Tarde, 13 set. 1900, p.1).

Na percepção dos jornais, jogos e dinâmicas esportivas seriam úteis para a robustez do corpo, ou seja, verdadeiras experiências proveitosas para a saúde, forjando corpos fortes para diversas demandas sociais. Nessa ambiência, conforme explorado por Ribeiro (1998), é provável que a emergência de um leque variado de associações e clubes esportivos na capital paranaense do início do século XX, junto ao aumento gradativo de imigrantes, artefatos e costumes de outros povos, notadamente de origem europeia, tenha contribuído de algum modo para uma melhor organização de outros esportes em Curitiba, entre os quais o futebol. Sobre este, assim um redator exclamou no *Diário da Tarde*:

No seio das sociedades de agora, ergue-se de novo, o par dessa evolução intelectual da mocidade, a compreensão nítida de quanto vale o desenvolvimento físico do homem, seguindo de perto o seu desenvolvimento moral, para que bem possa desempenhar a missão que o meio social lhe impõe e os deveres cívicos de cidadão e patriota (Diário da Tarde, 26 out. 1909, p.1).

É evidente o destaque dado à ligação do futebol com um discurso que reforça a relação das práticas esportivas às ideias de progresso da nação. A nota é nítida em visualizar o desenvolvimento físico em geral como um meio de educação cívica, moral e social da mocidade, contribuição fundamental para a construção de uma sociedade brasileira forte. As observações jornalísticas reforçam os êxitos que o futebol começava a alcançar:

De todos os esportes modernos, o que atualmente mais tem agradado é indubitavelmente o *football*, como tivemos ocasião de apreciar anteontem no *ground* do Jockey Club. Apesar de estarmos no inverno, o tempo proporcionou à festa um belíssimo domingo. Desde a manhã, notava-se nas ruas certo movimento festivo.

Quem lançasse ao meio-dia as vistas sobre as arquibancadas do Jockey-Club, notaria por certo o movimento extraordinário da assistência aguardando com ansiedade o começo do *match* entre os *foot-ballers* (A República, 14 jun. 1910, p.1).

Notemos que a partida futebolística retratada na fonte ocorreu nas dependências do Jockey Club do Paraná. A adaptação do centro da pista – segundo Souza (2014), uma área ociosa – possibilitava o ajuste em um campo de futebol. Ainda segundo o autor, o hipódromo em Curitiba também foi o espaço de difusão inicial do esporte bretão.

Notemos que os discursos de caráter médico-sanitário, preocupados com os cuidados e a melhoria do corpo aos quais os novos esportes e divertimentos físicos se vinculavam, ganharam uma narrativa de serem fundamentais para o desenvolvimento do país. Alinhados, portanto, a ideais de fortalecimento da mocidade em prol da robustez da nação, essas dinâmicas demonstraram em Curitiba ser úteis e agradáveis experiências para a modernização da cidade e dos costumes da população, sobretudo em relação à difusão de hábitos preocupados com revigoramento corporal.

## Considerações finais

Na virada do século XIX para o XX, as condições de saúde em Curitiba refletiam os desafios comuns enfrentados por outras cidades brasileiras. Epidemias de doenças infecciosas, como a febre amarela, a varíola e a cólera, eram frequentes, em razão das condições insalubres de vida nas áreas urbanas. A falta de infraestrutura básica, como abastecimento de água e saneamento adequado, contribuía para a propagação dessas doenças.

Nesse cenário, os divertimentos físicos e esportivos se materializam como uma rica oportunidade de intervenção urbana de dupla ordem: construção de espaços públicos e difusão de hábitos considerados saudáveis. Portanto, ao explorarmos as experiências esportivas e recreativas físicas promovidas em Curitiba, torna-se evidente que esses momentos de entretenimento demonstram as marcas dos ideais de urbanidade e saúde que se conformavam na capital paranaense.

Foi possível concluir que, em Curitiba, um discurso progressista e utilitário legitimou os entretenimentos físicos como importantes contributos para a cidade e os costumes da população, especialmente por visualizar essas dinâmicas como benéficas para a saúde por meio do fortalecimento corporal da juventude. Por outro lado, era essa mesma narrativa que reduziria outras atividades como menos úteis e interessantes. Práticas que não se enquadrassem nessas características teriam dificuldade em sustentar sua utilidade na esfera social.

Nessa perspectiva, considera-se que a relação entre esportes e saúde pública é dinâmica, refletindo as mudanças sociais e sanitárias do período estudado. No caso da capital paranaense, observa-se que a crescente valorização da prática esportiva e de atividades físicas é um meio de promover a saúde do corpo, bem como de fortalecer os valores morais. Essas práticas deveriam ser acompanhadas de posturas afáveis. Ideias como autocontrole, determinação, respeito pelas regras e espírito de equipe eram consideradas fundamentais não apenas para a jornada recreativa, mas também para todas as outras esferas da sociedade.

Valerá realizar novas inquirições acerca dos entretenimentos físicos e esportivos e sua proximidade com os discursos de saúde pública, tanto na capital paranaense quanto em outras localidades. Essas investigações, certamente, ajudarão a compreender as especificidades da estruturação dessas iniciativas e a lançar novos e originais olhares para a história da saúde, dos divertimentos, dos esportes e das cidades.

## Data Availability

Não estão em repositório.

## NOTAS

1 Devemos reforçar a aproximação com a noção desenvolvida neste artigo de que a modernização em Curitiba, conforme Lopes (2002), significava o conjunto de mudanças em curso, fossem elas de estruturas ou atitudes (em ambas, a emergência esportiva está presente), o que evidencia, portanto, que os esportes puderam direcionar para a modificação e a construção de novas atitudes/experiências, inclusive, aquelas relacionadas a ideias de hábitos considerados saudáveis.

2 Sobre o uso do termo “divertimento”, estamos alinhados com Santos (2017), que compreende o sentido e o significado da expressão em solo nacional. Segundo a autora, divertimento pode significar diversificadas atividades que têm em comum a potencialidade de causar sentimentos e expectativas, em uma relativa oposição a sério, rígido e severo. São, portanto, experiências que podem provocar alegria, prazer, euforia e regozijo – um verdadeiro leque de sensações, sobretudo para o espectador.

3 O respectivo termo tem o sentido e significado de vivência e experimentação urbana. Conforme aponta Menezes (2009), com o surgimento dos espaços públicos de diversão se formulam as figuras dispostas a usufruir dessa cena de rua. Assim, o *flâneur* constitui-se como ator social e elemento central da urbanização.

4 Nilo Cairo da Silva foi um dos mais importantes intelectuais do Paraná. Ganhou destaque como um dos mentores na criação da Universidade Federal do Paraná. Para mais informações sobre sua trajetória social, em especial a médica, ver Fraiz (2014).

5 Para uma intensa exploração das origens e do desenvolvimento da imigração britânica no Paraná, consultar o trabalho de Gilles (2010).
